# Framing the COVID-19 Pandemic Through a Family Lens: Results of a Qualitative Thematic Analysis

**DOI:** 10.1093/geroni/igab046.3212

**Published:** 2021-12-17

**Authors:** Lauren Gilbert, Omolola Adepoju, LeChauncy woodard, Daniel Howard

**Affiliations:** 1 University of Houston College of Medicine, Houston, Texas, United States; 2 Humana Integrated Health System Sciences Institute, Houston, Texas, United States; 3 Texas A&M University at College Station, Texas A&M University at College Station, Texas, United States

## Abstract

Background: The growing proportion of older adults in the U.S. population tends to be most vulnerable to the effects of natural disasters such as pandemics. To date, little has been done to counteract the impacts of public health emergencies and disasters on the aging populations, particularly in African American and Latinx communities. Methods: We administered a survey to community-dwelling minority older adults, 55+, in the Houston metroplex, between 11/2020 and 01/2021. The survey assessed how the COVID-19 pandemic has impacted minority older adults. This thematic analysis focused on open-ended questions regarding daily health management, biggest health concerns, and personal experiences with COVID-19. Results: A total of 575 older adults completed the survey. The mean age was 69 years, 74% were female, 71% reported English as their primary language and 27% were college educated. Three main themes of COVID-19 related concerns emerged from the thematic data analysis: 1) Fear of contracting COVID-19 from family members and fear of passing COVID-19 on to family members. 2) Social needs, including prolonged isolation from family/friends to stay safe, obtaining basic necessitates such as food, medications, and transportation . (3) Personal experiences focused on COVID-19 cases, hospitalizations, and deaths of family/community members. Conclusions: These older minority adults framed their experiences and concerns regarding the COVID-19 pandemic through the lens of family and their community. Their personal relationships permeated their responses and demonstrate the importance of integrating a family lens into future disaster planning, response and recovery efforts for minority older adults.

